# Using Diffusion of Innovation Theory to Address Health of the Homeless in Hawaii

**DOI:** 10.3390/healthcare11212828

**Published:** 2023-10-26

**Authors:** Lisa Spencer, Anita Tanner, Kristina Lu

**Affiliations:** 1 Long-Term Care, University of Hawaii-West Oahu, Kapolei, HI 96707, USA; lisaspen@hawaii.edu; 2 General Public Administration, University of Hawaii-West Oahu, Kapolei, HI 96707, USA; atanner@hawaii.edu; 3 Health Care Administration and Public Administration, University of Hawaii-West Oahu, Kapolei, HI 96707, USA

**Keywords:** diffusion innovation theory, homeless in Hawaii, CORE, CAHOOTS, STAR, EOTD

## Abstract

Policy diffusion is the process in which an innovation is communicated over time through various channels among the members of a social system. It is a special type of communication, in that the messages are concerned with new ideas. Diffusion theory or diffusion of innovations is the theoretical framework utilized in this study to compare successful programs aimed at providing health care for the homeless population. This study examines the Crisis Outreach Response and Engagement (CORE) program in Hawaii and explains how the diffusion innovation theory and programs in other states can be used to develop policies and processes to successfully address the medical and crisis intervention needs of the homeless on O’ahu. The study also includes suggested recommendations and strategies for the CORE program.

## 1. Introduction

Disputes with law enforcement and emergency services personnel, often exposed on social media, have highlighted the need for programs that de-escalate citizens in crisis, particularly those who are homeless. A growing social movement is occurring whereby programs addressing health-related issues must be designed to ensure individuals receive proper care in times of crisis and that law enforcement and medical personnel have the necessary training, resources, and personnel to assist these individuals. Policy diffusion “is the process by which an innovation is communicated through certain channels over time among the members of a social system. It is a special type of communication, in that the messages are concerned with new ideas” [1]. Diffusion theory or diffusion of innovations is the theoretical framework utilized in this study to compare successful programs aimed at providing health care for the homeless population.

This study, using O’ahu as its geographic locale, will examine the Crisis Outreach Response and Engagement (CORE) program in Hawaii and how diffusion innovation theory and programs such as CAHOOTS in Eugene, Oregon, STAR in Denver, Colorado, and EOTD in Los Angeles, California, can be used, or in some instances duplicated, to develop policies and processes to successfully address the medical and crisis intervention needs of homeless people on O’ahu. The study will also include suggested strategies for the CORE program, based on outcomes from CAHOOTS, STAR, and EOTD, on their target populations.

### 1.1. Literature Review

#### Homeless Population on O’ahu

The annual O’ahu Homeless Point-in-Time Count (PIT) survey identifies the needs and characteristics of individuals and families experiencing homelessness and is the primary source of data informing stakeholders and affecting federal funding [2] On 22 January 2020′s PIT, there were 4448 individuals experiencing homelessness on O’ahu [2]. Of the total population, 47% were sheltered and 53% unsheltered, 84% were adults (69% single adults), and 16% were children (85% of these children being sheltered) [2]. Overall, when compared to other populations, Native Hawaiian and Pacific Islanders (NHPI) and Multiracial individuals made up the largest percentages of homeless individuals [2]. Additional characteristics of the PIT population are as follows: 24% adults were chronically homeless individuals, 10% were veterans, 12% were adults 60 years and older, and 4% were unaccompanied minors [2].

Inability to pay rent, substance use, job loss, arguments with friends and family, and loss of money were the most common self-reported primary causes for homelessness [2]. Domestic violence, mental health problems, physical developmental disability, and living with HIV/AIDS were also noted to be causes for homelessness [2]. For the purpose of this research study, the health and wellbeing of the homeless population on O’ahu is the focus; however, the demographics of the population affects health care access and the need for specific health services, including behavioral health, geriatric care, and care related to determinants of health such as access to shelters and transportation.

A 2018 interview study of 162 homeless individuals on O’ahu, conducted by Withy, Amoa, Andaya, Inada, and Berry, concluded that the homeless individuals studied were three times more likely than the general population on O’ahu to rate their health as fair to poor, despite having medical insurance (77%) or a regular health care provider (66%) [3]. Younger age and having dental insurance were factors that improved one’s self-assessment of their health [3]. Commonly reported barriers toward good health included financial factors, environmental challenges, and general discomfort with the health care system [3]. Clinical implications of the study were centered around awareness of the health needs of the homeless and the challenges and barriers faced, including following medical advice [3]. While there were several limitations to this study, the challenges and barriers for receiving health care by the homeless population indicated a need for addressing the social determinants of health, including economic stability, health care access and quality, and the social and community context (i.e., housing) [4].

Dr. Josh Green, an ER physician and Lt. Governor of the State of Hawai’I, provides facts and resources on Homeless in Hawaii on his official website, ltgov.hawaii.gov. Dr. Green writes, “Hawaii is currently facing one of the worst homeless epidemics in the country with the highest rate of homelessness per capita in the nation [5]. It is the number one priority of the Lieutenant Governor to work towards solutions for our homelessness crisis which has a devastating social and economic impact on our state” [5]. Dr. Green goes on to write, “Housing is healthcare” [5]. By housing homeless individuals, 43 to 73% of Medicaid costs drop and people’s overall health outcomes drastically improve, including addressing existing medical conditions, lowering levels of stress, and decreasing infections, exposure to the elements, and the heightened risk for injuries, in addition to providing a safe place to store prescriptions [5].

The three most basic necessities in life are food, water, and shelter. Shelter is a social determinant of health and a key component of wellbeing. Hawaii has begun to address “shelter” with programs and initiatives including master-planned tiny home communities for the chronically homeless (Kauhale), public/private partnerships to create new models for addressing chronic homelessness (Hawaii Homeless Healthcare Hui—H4), joint outreach centers, permanent supportive housing (Housing First), assisted community treatment, and a law requiring treatment for individuals deemed a danger to themselves or others or those decompensating due to severe mental illness or addiction [5]. The new initiative CORE, Crisis Outreach Response and Engagement, is a solution-oriented approach that focuses on medical, social, and mental health needs, bringing appropriate care to the homeless on the street [6].

## 2. Methodology

Data were collected by reviewing articles and websites related to the diffusion theory, diffusion of innovations, and successful policies aiding the homeless population: “Records, documents, artifacts, and archives constitute a particularly rich source of information about many organizations and programs” [7]. A review of public online records was conducted to determine the details of each program as well as the successes (and failures). The authors’ reviewed the literature to identify and explain a conceptual framework to utilize in a best practices approach. A search of the library’s online database was conducted using relevant terms related to diffusion theory and diffusion of innovations. A search of websites was also conducted to identify programs that aided in assisting the homeless population. This review also provided a greater understanding of each program and its applicability and adoption into O’ahu’s CORE program. By reviewing the process of diffusion of innovations, the authors were able to ascertain key components that would aid in the success of O’ahu’s CORE program.

### 2.1. Diffusion Innovation Theory and Institutional Theory

Finding viable solutions to social issues is often plagued with many problems. Costs, personnel, equipment, timing, sunk costs, and public perception are some of the issues that often prohibit a government from implementing a policy that could solve a local issue. Policy diffusion is the act taken by one government in adopting a successful policy previously enacted in another government. The act of adoption is the diffusion or spreading of the policy. According to Mintrom and Walker, as cited in Shipan, C. R., and Volden, C., “Policy innovation occurs whenever a government—a national legislature, a state agency, a city—adopts a new policy” [8,9]. Policy diffusion is the process by which an innovation is communicated through certain channels over time among members of a social system [1,10]. The first channel is innovation. Innovation is an “idea or practice that is perceived as new by an individual or other unit of adoption”. This innovation provides a lens into the viability of a policy [1]. Diffusion of policy does not mean that the innovation is new; however, it is new to the government that is adopting it [11]. The government adopting the policy has the advantage of viewing the success and shortcoming of the policy before adoption it in their own system. The next channel is communication. Communication is the method by which the information travels from one government to the next [11]. Innovations need champions, and champions will discuss their new idea with peers [10]. This encourages buy-in to adopt the innovation. Once knowledge of the innovation is discovered, the innovation is evaluated for success in an existing system. If success is determined, the policy is implemented. Time and social system are the last two channels. Time explains how the unit adopting the policy gradually changes as it accepts the innovation, and the social system describes the individuals engaged in problem solving [11]. Moving too quickly to adopt a policy could introduce problems not previously identified. As policy is incremental, adopting a new policy takes time, as changes need to be made to fit into the current system. Diffusion runs through the social system by influences to adopt the new innovation and the communication that flows within the system [11]. If media spreads awareness of the innovation to the community, more individuals would be persuaded of the importance of this innovation, which would lead to adoption of the policy [12]. Policy diffusion is necessary, as it relates to a viable health care policy to assist the homeless population on O’ahu; it reduces the risk associated with policy implementation, as stated above.

“When confronted with a problem, decision makers simplify the task of finding a solution by choosing an alternative that has proven successful elsewhere” [13]. Knowledge is an important aspect of diffusing theory. Individuals must become aware of the policy and its success. If the policy is perceived to be successful, then a decision is made to adopt it. Cities are more inclined to implement a policy if it has already proven its success. Furthermore, policy diffusion is a kind of “social change, defined as the process by which alteration occurs in the structure and function of a social system [13]. When new ideas are invented, diffused, and are adopted or rejected, leading to certain consequences, social changes occur” [1,10].

### 2.2. Institutional Theory and Isomorphism

In sociology, an isomorphism is the resemblance of the processes of one organization to the processes of another. Organizations are socially rewarded by legitimacy, resources, and survival based on their acceptance of coercive, mimetic, normative institutional pressures, which lead to the diffusion of a policy and/or program [14]. Organizations tend to model themselves after similar organizations in their field that they perceive to be more legitimate or successful. We will focus our efforts on the first two, coercive isomorphism and the mimetic process. Coercive isomorphism results from both formal and informal pressures exerted on organizations by other organizations upon which they are dependent and by cultural expectations in the society within which organizations function [14]. In relation to our study, the need to have a more efficient means to aid the homeless in receiving health care has reached a point in which medical personnel and the community are seeking ways to improve upon the system of utilizing the emergency room for routine care. Thus, there is a need to diffuse policies from other cities to improve upon the services provided on O’ahu. Furthermore, mimetic processes or modeling states that uncertainty is a powerful force that encourages imitation; therefore, organizations may model themselves on other organizations [14]. Since modeling can be utilized as a response to uncertainty, the authors provide recommendations for the CORE program to adopt [14].

Policy diffusion is being utilized by many cities around the United States in finding a viable solution to serve our homeless population in times of non-emergent crisis. Oahu has recently adopted the Crisis Outreach Response and Engagement (CORE) model, which shares features of Crisis Assistance Helping Out On The Streets (CAHOOTS) in Eugene, Oregon, and the Support Team Assisted Response (STAR) in Denver, Colorado. Los Angeles also has a similar program, The Emergency Outreach and Triage Division (EOTD), which “oversees and monitors services to individuals experiencing mental health crisis” [15]. A summary of the literature on diffusion theory is presented in Table 1.

## 3. Discussion and Results

There are a few cities that have been identified as having very successful programs aimed at assisting the homeless population in health care needs. Crisis Assistance Helping Out On The Streets (CAHOOTS) in Eugene, Oregon, the Support Team Assisted Response (STAR) in Denver, Colorado, and the Emergency Outreach and Triage Division (EOTD) in Los Angeles, California, have each identified key successes within their programs. Table 2 highlights these successful programs and CORE.

The success in these programs led to reduced calls to 911 emergency services and trips to the emergency room, as noted below. In addition, the success of these programs, in particular the CAHOOTS program in Eugene, Oregon, received notable attention from other cities wanting to emulate their policy after they received media attention for service provided to the homeless.

### 3.1. Crisis Assistance Helping out On The Streets (CAHOOTS): Eugene, Oregon

Eugene, Oregon, started the Crisis Assistance Helping Out On The Streets (CAHOOTS) program in 1989. This mobile crisis intervention program provides various types of social services including crisis counseling and transport to individuals who are mentally ill, intoxicated, or disoriented, in addition to other non-emergency medical services [16]. CAHOOTS diverts calls from the police after it is determined their services are more apt for the situation. CAHOOTS works with the City of Eugene and is dispatched via the same system as EPD and Eugene Springfield Fire (ESF) and manages calls normally not handled by law enforcement.

The CAHOOTS program has evolved into a very successful program, with many city governments seeking to emulate or diffuse their program. For the program to be successful, it relies on three important factors: (1) an excellent network in the community; (2) trust in the people it serves; (3) culture that is built on caring and compassion to assist those in need [17]. Through the years, the program has struggled, as with most city programs, with funding. CAHOOTS emphasizes a need for programs to be well funded, as well as encouraging programs to prioritize paying their employees a living wage [18]. CAHOOTS costs about USD 2.1 million and is funded by the Eugene Police Department and various grants [18].

The success of CAHOOTS has been identified not only in the individuals they serve, but also it is able to respond to emergency calls normally handled by law enforcement and helps with transportation of patients for non-emergent care and mental health issues. In this way, CAHOOTS saved about USD 14 million in emergency medical costs in 2019 [18].

### 3.2. Support Team Assisted Response (STAR): Denver, Colorado

Similar to CAHOOT in Eugene, Oregon, the Support Team Assisted Response (STAR) program responds to low-risk calls by working in conjunction with clinicians and Denver paramedics or emergency medical technicians to provide medical assessment, crisis intervention, de-escalation, transportation, and resource connection for community members in need [19]. The team aids in assisting individuals experiencing mental health issues, poverty, homelessness, behavioral health crises, public health needs, and substance abuse [19].

The City and County of Denver conducted an evaluation of the STAR program after the 6-month pilot period. The data revealed that the STAR program was meeting its stated goals [20]. Realizing a larger data set would be needed to completely evaluate the program, the initial findings were very positive, even providing suggestions for future studies. The data also revealed that it was important to properly identify the call types for the team to handle and the necessity to construct a design tree for the assignment of those calls [20]. Blankets, cleaning supplies, food, and clothing were items found to be needed to assist individuals, and vans should be equipped with a wheelchair lift to allow for greater services to those unable to ambulate [20].

### 3.3. Emergency Outreach and Triage Division (EOTD): Los Angeles County, California

The Emergency Outreach and Triage Division (EOTD), part of the Emergency Outreach Bureau (EOB) of Los Angeles County’s Department of Mental Health, oversees and monitors homeless individuals experiencing mental health crises. Homeless Outreach Teams (HOT) are dedicated to assisting these homeless individuals at risk for incarceration or involuntary psychiatric hospitalization, often due to their mental illness [15]. EOB personnel respond in conjunction with law enforcement officers to calls involving mentally ill, violent, or high-risk homeless individuals [15].

Unsheltered homeless individuals are less likely to access health care and support services than those who are sheltered, a claim made by The Homeless Policy Research Institute (HPRI). The purpose of these organizations and agencies is to create outreach strategies to identify homeless populations and connect them to available services and housing resources [21]. Funding for these outreach efforts comes from a countywide sales tax for homeless services and prevention programs [21]. HPRI notes that homeless outreach programs are associated with increased service utilization, improved mental health, and substance use outcomes and are successful in improving health outcomes if tailored to specific populations (i.e., veterans) [21]. Some of the barriers to homeless outreach experienced by HOT personnel included distrust of outreach workers and service providers and a lack of racial and ethnic diversity in outreach and engagement practices [21]. Recommended best practices and strategies for homeless outreach programs included (1) multidisciplinary outreach teams that include people with lived homeless experiences that represent a diverse combination of roles and areas of expertise; (2) coordinated collaboration between agencies conducting outreach (i.e., homeless service providers and nontraditional partners); and (3) developing trust with clients through techniques like warm hand-offs [21]. Developing a countywide outreach system that leverages existing outreach efforts and involves different levels of government and community organizations, including data gathering and dissemination, is necessary to ensure that efforts are not duplicated or that people who need services are not missed [21].

An example of best practices and a recommended strategy for O’ahu’s homeless service providers is The People Concern. This is one of the largest social service agencies in Los Angeles County. It is a provider and advocate for homeless individuals and domestic violence victims [22,23]. The staff, volunteers, community, and outreach teams work together to address the effects of homelessness, poverty, mental and physical illness, abuse, and addiction and provide homeless individuals with links to resources and services that can help them be housed, healthy, and safe [22,23]. Currently, there are four LA outreach teams focused on addressing the most vulnerable unsheltered homeless, and in some situations, moving individuals from the streets to permanent housing [22,23]. The People Concern provides a multifaceted, fully integrated, and team approach to assisting individuals with outreach, temporary and permanent housing, mental and health services, domestic abuse services, and wellness programs [22,23].

### 3.4. Crisis Outreach Response and Engagement Program (CORE): Honolulu, Hawaii

The Crisis Outreach Response and Engagement (CORE) program, launched in October 2021 on O’ahu, is designed to help homeless people find transitional or permanent housing and navigate the obstacles homeless people face, including receiving health care. CORE dispatches a team of professionals, including social workers, EMTs, and community health workers, to nonviolent, homeless-related emergency calls, removing the Honolulu Police Department (HPD), Honolulu Fire Department (HFD), and Department of Emergency Services (EMS) from the process [24]. In one instance, the CORE team spent many hours navigating obstacles faced by a homeless Navy veteran but was finally able to connect him with the Veterans Affairs clinic who provided additional benefits so he could qualify for long-term foster care. According to Dr. Jim Ireland, EMS Director overseeing the CORE program, CORE aims to break the traditional EMS cycle of getting people off the street and putting them in a hospital and then back on the streets again [24]. CORE keeps a database that tracks each patient and outreach workers conducting walk-throughs in the neighborhoods where homeless people are to build connections, gather personal information, and relay the information back to CORE for further follow-up. According to Dr. Ireland, CORE’s concept was developed on ideas borrowed from some mainland programs [24]. CORE’s central role is focused on crisis, outreach, response, and engagement. An amount of USD 3.5 million was originally granted in funding from the COVID-19 American Rescue Plan Act but has increased to USD 5 million, allowing for expanded outreach to Windward O’ahu [25]. As the program just launched in October 2021, little data are available for the evaluation of the effectiveness of the program. However, initial numbers have shown that the CORE personnel serviced approximately 150 individuals [26].

## 4. Policy Diffused

The CAHOOTS, STAR, and EOTD programs were developed to help alleviate the case load placed on emergency services, law enforcement, and emergency room personnel for low-risk calls. Many times, individuals called 911 for assistance when the situation was not emergent. Each of these calls increased the costs associated with time, personnel, and funding. The utilization of these alternative programs shows that health care, when working in conjunction with law enforcement or emergency services, can aid in the increase in services to the homeless populations, while also alleviating much of the strain on law enforcement and emergency services personnel and addressing social determinants of health for people who are homeless. A key factor in diffusing policy is that decision makers are able to see the consequences of the policy before adoption. By diffusing policies and programs implemented in Eugene, Denver, and Los Angeles, the CORE program in Honolulu can benefit from lessons learned and proven statistical results, as shown through savings both in resources and financially.

### 4.1. Implications, Recommendations, and Strategies

#### 4.1.1. Implications

Policy makers rely on examples and insights from those who have experimented with policies in the past; therefore, understanding policy diffusion is crucial to understand policy advocacy and policy change [9]. Policy diffusion is one method for governments to utilize to implement programs that have been successful in other states. The CORE program on O’ahu would like to realize growth and expand its outreach; therefore, by diffusing policy, the CORE program will have an opportunity to limit risk and other externalities associated with growth.

#### 4.1.2. Recommendations and Strategies

Stakeholders of CORE can use ongoing data and results from programs such as CAHOOTS, STAR, and EOTD to make improvements to their program and, in some cases, address problems before they arise. Looking at the diffusion channels of innovation, communication, time, and social systems, CORE can implement viable and successful policies and programs and reduce the risks of failures to the program. Suggested strategies for CORE stakeholders are gained through policy diffusion. Table 3 contains a summary of the suggested strategies.

#### 4.1.3. STAR Strategies

The strategies included in the STAR program that can be utilized in CORE are as follows:

1. Understand the population served, as it will ensure the program is culturally relevant and responsive.
  - Culturally competent training should be specific to the population(s) served (i.e., NHPI, veterans, elderly, chronically homeless, and unaccompanied minors); recognize personal biases, prejudices, and stereotypes toward these populations/people; and teach the communication skills needed to address homeless people’s concerns and negotiate effectively and collaboratively to optimize health care outcomes.
2. Conduct a cost–benefit analysis to provide a clearer picture of the return on investment for government (federal, state, and local), health and human service providers, and the community.
  - Since CORE has not reached its first-year milestone, only funding data are available to the public. It will be important to see how the USD 3–5 million will be allocated to CORE, and especially the outcomes based on the spending (ROI). Specifically, was CORE able to meet the medical, social, and mental health needs of the homeless they served?
  - The initial funding for CORE was granted from the COVID-19 American Rescue Plan Act. Using the cost–benefit analysis model to seek a more permanent stream from the state legislature to ensure the program’s growth and longevity is needed. Conversely, and to follow a policy diffusion approach, funding through the Honolulu Police Department could be sought, or perhaps a combination of public and private partnerships.

#### 4.1.4. CAHOOTS Strategies

The strategies included in the CAHOOTS program that can be utilized in CORE are as follows:

3. Ensure robust human services and health care networks to address the needs of the homeless community.
  - A task force may be needed to explore human services and health care networks available to help the homeless populations and to provide opportunities for collaboration and the incorporation of programs and services as part of CORE’s services. There may be opportunities to develop alternate strategies through collaborative efforts.
  - According to the National Library of Medicine, there are four elements a program can utilize to ensure a robust network (Institute of Medicine): (a) frequent and consistent interaction by agencies involved; (b) a coordinated effort to link clients with needed services by providers; (c) a proactive approach in outreach efforts; (d) a review of internal and external resources to include funding, use of volunteers if appropriate, and the ability of homeless individuals to access the established network.
4. Establish trust from the population served.
  - Mistrust of health care service providers, including emergency medical services personnel, may prevent people from accessing care, which increases health disparities and affects health complications and outcomes. Trust can be built through the education and training of CORE staff and hiring CORE staff that reflect the homeless population—someone who looks like me and/or has been through what I have been through or am going through.
5. Build a community culture of care and compassion, supporting a helpful and healthy response to struggling community members.
  - Build a robust website that includes links to resources to meet the needs of the homeless people (i.e., housing, transportation, health care, social service resources, and government benefits).
  - Have available written materials (i.e., brochures) for those people who may not have access to the website. Have community health workers, homeless organizations, and others distribute these informational brochures as they are conducting business.

#### 4.1.5. EOTD Strategies

The strategies included in the EOTD program that can be utilized in CORE are as follows:

6. Develop specialized community-based programs in other areas of O’ahu (and the neighbor islands) that target the homeless population and specialized full-service partnership programs such as Hawaii Homeless Healthcare Hui (H4).
  - With such a diverse homeless population, programs that target specific groups may not work in other communities or with other groups of homeless individuals. Also, with Hawaii being made up of several islands, programs may not have funding or resources to offer their services outside of the main island of O’ahu. Developing programs that provide safe and functional environments for homeless individuals also alleviates financial and medical strains on emergency rooms and health systems and brings mental health professionals and social workers to the people, instead of requiring the people to navigate the health care system to receive services.
7. Increase the portfolio of housing resources.
  - Prioritizing housing opportunities, having those difficult discussions about housing in Hawaii, and moving people from the streets into safe housing should be a priority.
  - Homeless shelters, transitional housing, affordable housing, public and subsidized housing, affordable rentals and supportive housing, and housing assistance are opportunities to provide housing for homeless people. Finances and financial support are factors that make finding affordable housing for homeless people difficult.
  - Taking a holistic approach, the focus should be on affordable housing and include focused health and human service programs to qualifying residents, which would increase the rates of permanent housing placements.
8. Participate in collaborative efforts to end homelessness.
  - An Aloha Friday Conversation: Seizing affordable housing opportunities in Hawaii, on Hawaii Public Radio (2022) brought together representatives from the Hawai’i Budget and Policy Center, Hawaiian Community Assets, Hawaii Appleseed, the Governor’s Office on Homelessness, the Honolulu’s Tenants Union Organizing Committee, Partners in Care, the Institute for Human Services, Faith Action for Community Equity, Hawaiian Community Assets, and Permanently Affordable Living to discuss this topic of homelessness.
  - Other possible collaborators are as follows: City and County of Honolulu, Hawaii Public Housing Authority, Hawaii State Government, financial/banking industry, health care systems, the Institute for Human Services, housing development companies, real estate developers and companies, public and private organizations, representatives from homeless communities, and the community at large.
  - CAHOOTS credits much of its success to a collaborative effort. Realizing a mobile unit alone is not sufficient to support the myriad of needs of the homeless population; therefore, other social services should work in conjunction with the CORE unit to provide support for housing and mental and physical health needs.

## 5. Limitations of Study

There were several limitations in this study. Due to the location of O’ahu on an island and the cultural diversity of the homeless population, not all recommendations will be viable in other areas. The authors searched for specific keywords to gather relevant information pertaining to diffused theory as well as outreach programs aimed at homeless populations. Furthermore, this study was not comprehensive of all programs in all U.S. cities. The authors focused on successful program from cities located on the West Coast of the United States due to the similarity of climate and geographical proximity to the island of O’ahu. The purpose of this research was to shed light on homelessness and health care on O’ahu as well as provide some relevant successes of other organizations. Future research will include first-hand interviews with key stakeholders, including homeless community representatives, as identified in the recommendation section.

## 6. Conclusions

Policy diffusion is the process by which an innovation is communicated through certain channels over time among members of a social system [9]. Policy diffusion is one method that policy makers can use to find viable solutions to issues that arise [1,10]. The Crisis Outreach Response and Engagement (CORE) program on O’ahu, Hawaii, was designed with a focus on assisting homeless individuals with their medical and social needs. Using data and outcomes from similar programs such as CAHOOTS, STAR, and EOTD, Hawaii’s CORE stakeholders can continue to address the issue of homelessness and health care for Hawaii’s communities.

## Figures and Tables

**Table 1:** Diffusion theory/diffusion of innovations: review of literature.

Author(s)	Year of Publication	Key Points
Baybeck, B., Berry, W., and Siegel, D. [13]	2009	Finding a solution by choosing an alternative that has proven successful elsewhere. Policy diffusion is a kind of “social change, defined as the process by which alteration occurs in the structure and function of a social system.”
Berry F and Berry W. [11]	2009	Government adopting the new policy has the advantage of viewing the success and shortcoming of the policy before adoption in their own system. Channels of communication consist of innovation, communication, time, and social system.
DiMaggio, P. J. and Powell, W. W. [14]	1983	Isomorphism is the resemblance of processes of one organization to another. Coercive isomorphism results from both pressures exerted on upon which they are dependent and by cultural expectations in society. Mimetic processes states that uncertainty is a powerful force that encourages imitation.
Haider, M. and Kreps, G. L. [12]	2004	Media spreading awareness of the innovation will lead to the adoption of the policy.
Rogers, E. M. [1]	1995	Process of communicating innovation through channels. Provides a lens of the viability of the program.
Rogers, E. M. [10]	2002	Innovations need champions to discuss their new idea with peers. This encourages buy-in to adopt the innovation.
Shipan, C. R. and Volden, C. [8]	2008	Policy innovation occurs when a government adopts a new policy.
Shipan, C. R. and Volden, C. [9]	2012	Policy makers rely on examples and insights from those who have experimented with policies in the past.

**Table 2:** A summary of successful programs and CORE.

Author(s)	Year of Publication	Main Contents
**CAHOOTS—Crisis Assistance Helping Out on the Streets—Eugene, OR, USA**		
Eugene Police Department [16]	2019	CAHOOTS program described. How CAHOOTS is supported by the city. How services are accessed. How CAHOOTS is the primary responder providing valuable and needed resources to the community.
White Bird Clinic [17]	2020	CAHOOTS program described. Services provided. Contact information. Informational brochure. Funding and budget information. Link to the program analysis (2020). https://www.eugene-or.gov/DocumentCenter/View/56717/CAHOOTS-Program-Analysis
Legislative Analysis and Public Policy Association [18]	2020	Background and history of CAHOOTS. Operations information. Data on calls and responses by CAHOOT. Funding data. Contact information for cities and towns looking to duplicate the CAHOOTS model.
**STAR—Support Team Assisted Response—Denver, CO, USA**		
Denver: The Mile High City, The Denver Local [19]	2023	STAR program described. Who does STAR help? Operations data. Partners. 2022 mid-year report https://www.denvergov.org/files/assets/public/v/1/public-health-and-environment/documents/cbh/2022_midyear_starreport_accessible.pdf Advisory Committee information.
STAR Program Evaluation [20]	2021	Executive summary. Pilot area and nature codes. Data overview. Lessons learned. Recommendation for future growth and research.
**EOTD—Emergency Outreach and Triage Division—Los Angeles County, CA, USA**		
Homeless Policy Research Institute (HPRI) [21]	2020	Background. Literature review and data analysis. Research findings. Best practices and recommended strategies.
The People Concern [22]	2022	Description of the program. Organizational reports: 2021–2022 impact report http://www.thepeopleconcern.org/wp-content/uploads/2023/02/Impact-Report_Resized009.pdf
The People Concern [23]	2023	Outreach teams.
**CORE—Crisis Outreach Response and Engagement program—Honolulu, HI, USA**		
Ordonio C [24]	2021	News article on the CORE program.
Mizuno A [25]	2021	News article describing the CORE program.
Schaefers A [26]	2022	News article describing the CORE program.

**Table 3:** A summary list of strategies for CORE from STAR, CAHOOTS, and EOTD programs.

Program:	Strategies:
**STAR**	Understand the population served, as it will ensure the program is culturally relevant and responsive.
	2. Conduct a cost–benefit analysis to provide a clearer picture of the return on investment for government (federal, state, and local), health and human service providers, and the community.
**CAHOOTS**	1. Ensure robust human services and health care networks to address the needs of the homeless community.
	2. Establish trust from the population served.
	3. Build a community culture of care and compassion, supporting a helpful and healthy response to struggling community members.
**EOTD**	1. Develop specialized community-based programs in other areas of O’ahu (and the neighbor islands) that target the homeless population and specialized full-service partnership programs.
	2. Increase the portfolio of housing resources.
	3. Participate in collaborative efforts to end homelessness.

## Data Availability

Not applicable.

## Abbreviations

CORE	Crisis Outreach Response and Engagement program
CAHOOTS	Crisis Assistance Helping Out on The Streets
STAR	Support Team Assisted Response
EOTD	Emergency Outreach and Triage Division
PIT	Point in time
